# Cross-Sectional Unification on the Stress-Strain Model of Concrete Subjected to High Passive Confinement by Fiber-Reinforced Polymer

**DOI:** 10.3390/polym8050186

**Published:** 2016-05-11

**Authors:** Yu-Gui Cao, Cheng Jiang, Yu-Fei Wu

**Affiliations:** 1 School of Civil Engineering and Mechanics, Huazhong University of Science & Technology, Wuhan 430074, China; caoyugui@163.com; 2 Department of Architecture and Civil Engineering, City University of Hong Kong, Hong Kong SAR, China; yufei.wu@rmit.edu.au; 3 Department of Civil, Construction and Environmental Engineering, Marquette University, Milwaukee, WI 53233, USA; 4 School of Civil, Environmental and Chemical Engineering, RMIT University, VIC 3001, Melbourne, Australia

**Keywords:** fiber-reinforced polymer (FRP), concrete, confinement, stress-strain relationship, cross-sectional unification, modeling

## Abstract

The stress-strain behavior of concrete can be improved by providing a lateral passive confining pressure, such as fiber-reinforced polymer (FRP) wrapping. Many axial stress-strain models have been proposed for FRP-confined concrete columns. However, few models can predict the stress-strain behavior of confined concrete columns with more than two specified cross-sections. A stress-strain model of FRP-confined concrete columns with cross-sectional unification was developed in this paper based on a database from the existing literature that includes circular, square, rectangular and elliptical concrete columns that are highly confined by FRP jackets. Using the database, the existing theoretical models were evaluated. In addition, the ultimate stress and strain models with cross-sectional unification were proposed using two parameters: the cross-sectional aspect ratio and corner radius ratio. The elliptical cross-section can be considered as a rectangular one with a special corner radius for the model calculations. A simple and accurate model of the equivalent corner radius ratio for elliptical columns was proposed. Compared to the other existing models and experimental data, the proposed models show good performance.

## 1. Introduction

Fiber-reinforced polymer (FRP) is widely used in structural strengthening and retrofitting [1,2,3,4,5]. Various studies have been conducted on the mechanical performance of FRP-confined concrete columns, and many models for the stress-strain relationship, ultimate strain and ultimate stress have been proposed [6,7,8,9,10,11,12,13,14,15,16,17,18,19,20,21,22,23,24,25,26,27]. The basic principle of the FRP-confined concrete column is that the FRP can be activated by concrete lateral dilation under axial loading to provide the confining pressure that improves the axial strength and ductility. The existing research results show that the cross-sectional shape of concrete columns has a significant impact on the FRP lateral confining pressure [12,19,20,27,28,29]. Therefore, many researchers have proposed different stress-strain models and ultimate strain and stress models for different column cross-sections. However, very few unified models have been proposed that can calculate the stress-strain behavior of FRP-confined concrete columns with various cross-sections [13,19,27,28]. This issue often causes inconvenience in structural retrofitting design. In practical engineering, the common cross-sections of reinforced concrete (RC) columns are square, rectangular, circular and occasionally elliptical. Engineers have to select different models according to the different cross-sections. A rational model needs to be developed that can predict more cross-sectional shapes for easy use by engineers.

This paper aims to develop a cross-sectional unified stress-strain model that considers the variation of cross-sections from square/rectangular to circular/elliptical. The transformation of the cross-section is considered by adjusting the cross-sectional aspect ratio and the corner radius ratio. Because the strain-hardening case is more common than strain-softening, this paper focuses on the stress-strain behavior of concrete with strain-hardening (or with high confinement).

## 2. Existing Stress-Strain Models

**Figure 1:**
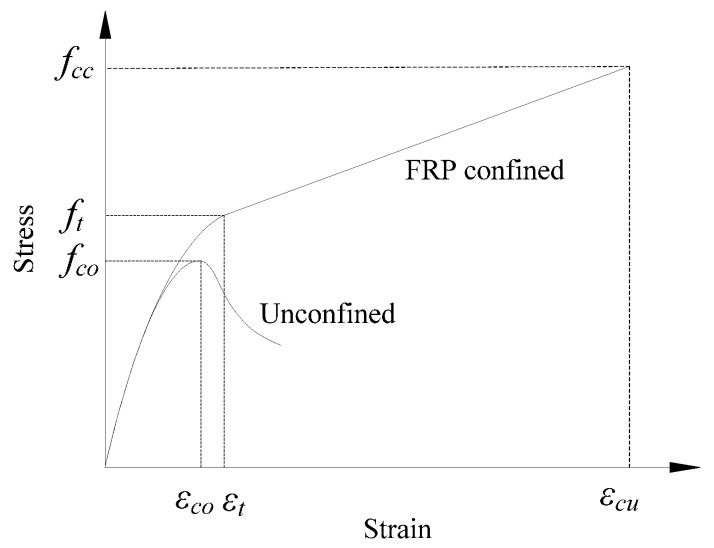
The typical stress-strain curve for FRP-confined concrete with strain-hardening.

To date, more than 90 stress-strain models have been proposed for FRP-confined concrete columns under axial loading [30,31]. Most of the stress-strain models for confined concrete with strain-hardening are divided into two parts by the transitional point (*f_t_*, ε*_t_*), as illustrated in Figure 1. The first part is a parabola curve before the transitional point, and the second part is a straight line after the transitional point. Some selected existing unified stress-strain models with different cross-sections are reviewed in this section. The details of the calculations in the discussed models are listed in Table 1.

### 2.1. Models by Teng’s Group

Teng’s group presented different stress-strain models and ultimate stress and strain models for different cross-sections [14,15,32,33]. All of the stress-strain models are divided into two parts by a transitional point and can be expressed in the following general forms:

(1a) $$\begin{array}{cc} f_{c}=E_{c}\varepsilon_{c}-\frac{\left(E_{c}-E_{2}\right)}{4f_{co}}\varepsilon_{c}^{2} & 0\leq \varepsilon_{c}\leq \varepsilon_{t} \end{array}$$

(1b) $$\begin{array}{cc} f_{c}=f_{co}+E_{2}\varepsilon_{c} & \varepsilon_{c}>\varepsilon_{t} \end{array}$$

where *E_2_* = (*f_cc_* − *f_co_*)/ε*_cu_*; *f_c_* is the axial stress at the axial strain ε*_c_*; ε*_t_* is the strain at the transfer point, which can be calculated as ε*_t_* = 2*f_co_*/(*E_c_* − *E_2_*); *f_cc_* and ε*_cu_* are the ultimate stress and strain, respectively (see Figure 1); *E_c_* and *f_co_* are the elastic modulus and strength of unconfined concrete, respectively; and *E*_2_ is the slope of the linear hardening part of the stress-strain curve or hardening slope. They used different expressions to predict different cross-sections (see Table 1). Similar to most related investigations, the ultimate stress (*f_cc_*) models were proposed based on an early work by Richart *et al.* [34] (*f_cc_*/*f_co_* = 1 + *k*(*f_l_*/*f_co_*) in Table 1). Different works propose different values for the coefficient *k* based on different approaches and databases. Lam and Teng suggested two in [33] for the *k* value before proposing 3.3 with a more systematic study in [14] for circular columns. For different cross-sections, they introduced different shape factors into the *k* value. Thus, different cross-sections have individual equations for the ultimate stress and strain. 

### 2.2. Model by Youssef et al.

Youssef *et al.* proposed a stress-strain model for circular and rectangular columns confined by FRP [28]. The model is listed as follows:

(2a) $$\begin{array}{cc} f_{c}=E_{c}\varepsilon_{c}\left[1-\frac{1}{n}\left(1-\frac{E_{2}}{E_{c}}\right){\left(\frac{\varepsilon_{c}}{\varepsilon_{o}}\right)}^{n-1}\right] & 0\leq \varepsilon_{c}\leq \varepsilon_{o} \end{array}$$

(2b) $$\begin{array}{cc} f_{c}=f_{o}+E_{2}\left(\varepsilon_{c}-\varepsilon_{o}\right) & \varepsilon_{o}\leq \varepsilon_{c}\leq \varepsilon_{cu} \end{array}$$

where $n=\frac{\left(E_{c}-E_{2}\right)\varepsilon_{o}}{E_{c}\varepsilon_{o}-f_{o}}$. In terms of the different cross-sections, Youssef *et al.* [28] suggested different formulas for the stress and strain at the transitional (*f_o_*, ε*_o_*) and ultimate (*f_cc_*, ε*_cu_*) points, which are listed in Table 1. 

### 2.3. Model by Hu and Wang

Hu and Wang [13] proposed a stress-strain model with two parts, which can be used to predict the circular, rectangular and square columns confined by FRP:

(3a) $$\begin{array}{cc} f_{c}=\frac{A\varepsilon_{c}}{1+B\varepsilon_{c}+C\varepsilon_{c}^{2}} & 0\leq \varepsilon_{c}\leq \varepsilon_{\text{ct}} \end{array}$$

(3b) $$\begin{array}{cc} f_{c}=f_{ct}+E_{2}\left(\varepsilon -\varepsilon_{ct}\right) & \varepsilon_{c}＞\varepsilon_{ct} \end{array}$$

where $A=E_{c}=4700\sqrt{f_{co}}$, $B=\frac{E_{c}}{f_{ct}}-\frac{2}{\varepsilon_{ct}}$, $C=\frac{1}{\varepsilon_{ct}^{2}}-\frac{E_{c}E_{2}}{f_{ct}^{2}}$, $E_{2}=\frac{f_{cc}-f_{ct}}{\varepsilon_{cu}-\varepsilon_{ct}}$; the functions of the transfer strain ε*_ct_* and stress *f_ct_* are listed in Table 1 with the ultimate stress and strain models. 

### 2.4. Model by Wei and Wu

By introducing two parameters (cross-sectional aspect ratio and the corner radius ratio), Wei and Wu proposed a unified stress-strain model (Equations (4a) to (4b)) for FRP-strengthened circular, rectangular and square columns [19]. The ultimate and transitional stress and strain models (listed in Table 1) were proposed and verified with a large database.

(4a) $$\begin{array}{cc} f_{c}=E_{c}\varepsilon_{c}+\frac{f_{o}-E_{c}\varepsilon_{o}}{\varepsilon_{o}^{2}}\varepsilon_{c}^{2} & 0\leq \varepsilon_{c}\leq \varepsilon_{o} \end{array}$$

(4b) $$\begin{array}{cc} f_{c}=f_{o}+E_{2}\left(\varepsilon_{c}-\varepsilon_{o}\right) & \varepsilon_{o}\leq \varepsilon_{c}\leq \varepsilon_{cu} \end{array}$$

**Figure 2:**
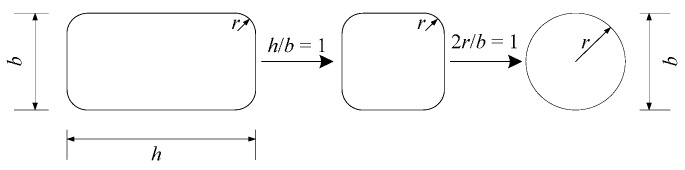
Cross-sectional unification by Wei and Wu [19].

Wei and Wu [19] used two parameters: cross-sectional aspect ratio *h/b* and the corner radius ratio 2*r/b*. These two parameters unify the circular and square cross-section: when *h*/*b* = 1 and 2*r/b =* 1, the concrete column is circular; if *h*/*b* = 1 and 2*r/b ≠* 1, the concrete column is square with a corner radius; and when *h*/*b* *≠* 1 and 2*r/b ≠* 1, the concrete column is rectangular with a certain corner radius, as illustrated in Figure 2. Using these two parameters, Wei and Wu [19] proposed a general stress-strain model for circular, square and rectangular columns. However, elliptical columns cannot be directly defined by these parameters of *h/b* and 2*r/b*.

## 3. Experimental Database

A large database for the stress-strain curves is built in this work. The data selection criteria are as follows: (1) the FRP sheets are unidirectional in the lateral direction; (2) the mechanical properties of the FRP sheets are obtained from coupon tests; and (3) the concrete strength, *f_co_*, is selected as the cylinder strength of unconfined concrete. The database contains 296 stress-strain curves of FRP-confined concrete columns in total, with 181 circular columns [25,35,36,37,38,39,40,41,42,43,44,45], 23 elliptical columns [32,39], 68 square columns and 24 rectangular columns [6,15,40,46,47,48,49,50,51,52]. The concrete strength ranges from 18.3–85.6 MPa. The details of the database are listed in the Table 2.

Two indexes (Equations (5) and (6)), which are sensitive to deviations between the modeling and test data [23,24,25,53,54], are used to evaluate the existing and proposed models in this work: (1) average value (*AV*); (2) integral absolute error (*IAE*).

(5) $$AV=\frac{\sum_{1}^{n}\frac{Theo_{i}}{Expe_{i}}}{n}$$

(6) $$IAE=\frac{\sum_{1}^{n}\left|Theo_{i}-Expe_{i}\right|}{\sum_{1}^{n}\left|Expe_{i}\right|}$$

in which *Theo*_i_ is the theoretical results, *Expe*_i_ is the experimental data and *n* is the experimental data number. For the index *AV*, the nearer the *AV* value is to one, the more accurate the model is; the lower the *IAE* value is, the better the results are.

## 4. Stress-Strain Modeling

### 4.1. General Mathematical Model

**Figure 3:**
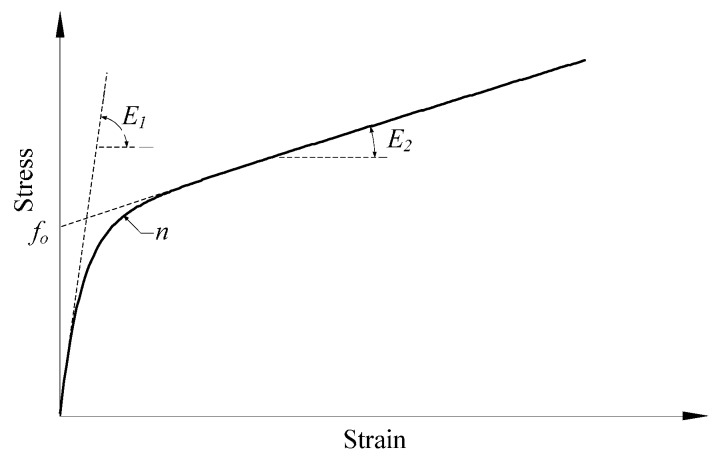
Stress-strain relationship (Equation (7)).

A monotonic continuous expression with four parameters (*E*_1_, *f_o_*, *E_2_* and *n*) proposed by Zhou and Wu [55] can be used to fit the stress (*f_c_*)-strain (ε*_c_*) behavior of the FRP-confined concrete column:

(7) $$f_{c}=[(E_{1}\varepsilon_{n}-f_{o})e^{-\frac{\varepsilon_{c}}{\varepsilon_{n}}}+f_{o}+E_{2}\varepsilon_{c}](1-e^{-\frac{\varepsilon_{c}}{\varepsilon_{n}}})$$

where *E*_1_ is the initial stiffness for the stress-strain curve, *f_o_* is the intersection stress value between the asymptotic line and the y axis (see Figure 3), *E_2_* is the hardening stiffness or the slope of the asymptotic line of the stress-strain curve after the transfer point and ε*_n_* = *n ×* ε*_o_*, ε*_o_* = *f_o_*/*E*_1_, where *n* is a parameter satisfying 0 < *n* ≤ 1 that controls the curvature of the transfer part. Equation (7) is illustrated in Figure 3. The parameters of *f_o_*, *E*_1_, *n* and *E*_2_ can be obtained by numerical regression on experimental stress-strain curves using the mathematically-continuous Equation (7) [21,43,55].

### 4.2. Cross-Sectional Analysis

**Figure 4:**
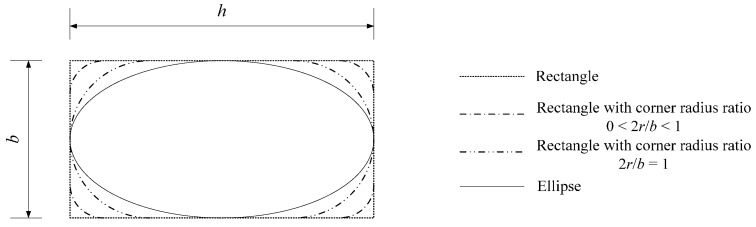
The details of rectangular and elliptical cross-sections.

Wei and Wu [19] have perfectly related the transition from rectangular to square and circular cross-sections. However, the elliptical cross-section cannot be considered in their model due to the complex geometric relationship. Geometrically speaking, ellipses are related to rectangles. An ellipse can be inscribed in a rectangle, as shown in Figure 4. For column cross-sections, the rectangular column should be ground with a corner radius before FRP wrapping to achieve more confinement efficiency (dotted dashed line in Figure 4). Similarly, the inscribed elliptical cross-section can be obtained from the rectangular cross-section by grinding. Nevertheless, the grinding for an elliptical cross-section is different than that for normal rectangular ones. Every point at the side has an individual curvature without a unified corner radius. Such a transformation from a rectangle to an ellipse leads to the unification of the column cross-sections.

For FRP-confined concrete columns, the relationship between the ellipse and rectangle can be quantified by the equivalent confinement efficiency. The elliptical cross-section can be considered the rectangular one with a special corner radius for calculations. For quantitative analysis, an equivalent corner radius, *r_e_*, can be introduced to equivalently form an elliptical to rectangular cross-section. Furthermore, the aspect ratio, *h*/*b*, can affect this transformation because a greater aspect ratio can cause a greater difference on the shape between the ellipse and rectangle with a corner radius. The relationship between *h*/*b* and 2*r_e_*/*b* is assumed to be nonlinear at first. When *h = b* or *h*/*b* = 1, a rectangle becomes a square and an ellipse becomes a circle. In this case, 2*r_e_*/*b* should be equal to one. Therefore, the equivalent corner radius ratio model can satisfy:

(8) $$\frac{2r_{e}}{b}=1-\alpha {\left(\frac{h}{b}-1\right)}^{\beta }$$

where α and β are coefficients that need to be determined. 

### 4.3. Parameters in Modeling

All of the parameters in Equation (7) can be obtained from nonlinear numerical regression on the stress-strain curves of the specimens in Table 2. The parameter considerations are listed as follows.

#### 4.3.1. Elastic limit *f_o_*

**Figure 5:**
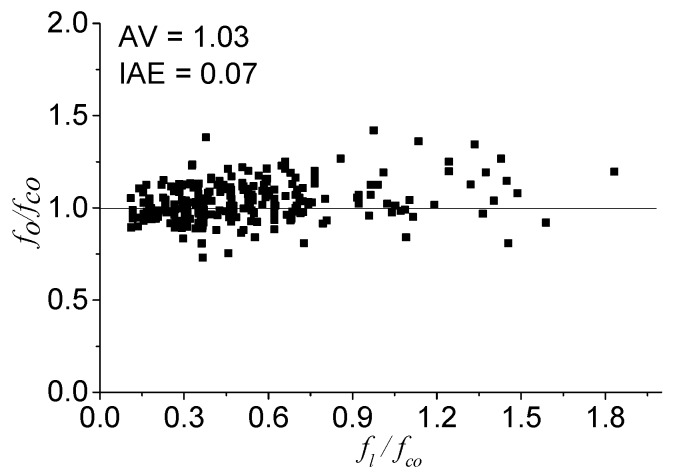
The performance of *f_o_*.

*f_o_* is believed to be the limit value of the elastic stage of the stress-strain relationship [43]. After regression, the *f_o_* values obtained from the 273 stress-strain curves are shown in Figure 5. All of the values of *f_o_*/*f_co_* are distributed between 0.8 and 1.4, regardless of the confinement. The *AV* and *IAE* values for the *f_o_* are 1.03 and 0.07. In terms of concrete under high confinement, which is the research subject in this work, the *f_co_* value can be taken as *f_o_* for the sake of simplification [14]:

(9) $$f_{o}=f_{co}$$

#### 4.3.2. Initial modulus *E*_1_

*E*_1_ is the initial tangential stiffness modulus of the stress-strain curve. Similarly, *E*_1_ values are also obtained from the regression results using Equation (7). The elastic modulus of concrete, *E_c_*, is believed to be proportional to the *f_co_*^0.5^, such as 4730 *f_co_*^0.5^ [56]. However, *E*_1_ is the initial tangential modulus, which is shown to be greater than *E_c_* [21]. Therefore, the relationship between *E*_1_ and *f_co_* can be regressed using *E*_1_ = *k*_1_*f_co_*^0.5^:

(10) $$E_{1}=5573{f_{co}}^{0.5}$$

**Figure 6:**
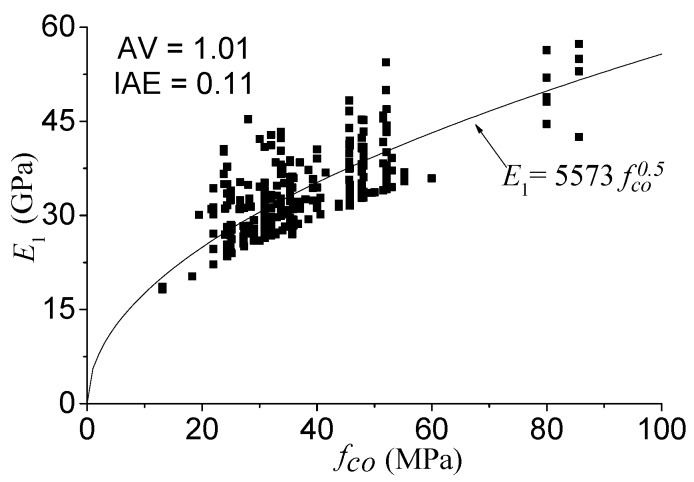
The performance of the *E*_1_ model.

The performance of Equation (10) is shown in Figure 6; the evaluation index values of *AV* and *IAE* are 1.01 and 0.11, respectively, which means that Equation (10) can be used to accurately calculate *E_1_* values.

#### 4.3.3. Parameter *n*

Parameter *n* does not significantly affect the stress-strain curve and is only related to the transition zone [21,43,55]. The value of *n* is between zero and one. The regression results of the *n* value range from 0.6–1. The confinement ratio does not significantly affect the parameter *n* value. For the sake of simplification, the average value is used to define the *n* value: *n* = 0.76.

#### 4.3.4. Hardening modulus *E*_2_

In most existing ultimate stress and strain models, researchers consider the confining pressure *f_l_* (Equation (11)) to determine the stress-strain behavior [14,15,19,20,27]. Such a determination is not very rational or reasonable. The confining pressure *f_l_* is the lateral pressure value when the FRP is at failure, and the ultimate point of the stress-strain curve is controlled by the FRP ultimate strain. Therefore, using either *f_l_* or confining stiffness *E_l_* (Equation (12)) is acceptable to determine the ultimate stress and strain of confined concrete. However, the hardening stiffness *E*_2_ (calculated by Equation (18)) would be more rational to be related to *E_l_* or the confinement stiffness ratio *E_l_*/*f_co_* [45,57]. Therefore, the confinement stiffness *E_l_* and rupture strain of FRP ε*_f_* are used to model the ultimate stress and strain of confined concrete in this work.

(11) $$f_{l}=\frac{2E_{frp}t\varepsilon_{f}}{b}$$

(12) $$E_{l}=\frac{2E_{frp}t}{b}$$

where *b* is the diameter of a circular column or the side length of a square column or the shorter side length of a rectangular column [19]; *t* is the thickness of wrapped FRP.

The concrete strength and cross-sectional shape also affect the ultimate stress and strain [15,19,20,23,58,59]. The ultimate stress and strain model proposed by Wei and Wu has the advantages of continuous expression, accurate prediction and including different parameters simultaneously [19,60]. Therefore, more reasonable ultimate stress and strain models are proposed by modifying the Wei and Wu model [19] using Equations (13) and (14) for modeling in this work, which can be used to calculate the ultimate stress of FRP-confined circular, square, rectangular and elliptical cross-sections.

(13) $$\frac{f_{cc}}{f_{co}}=1+n_{1}{\left(\frac{E_{l}}{E_{c}}\right)}^{n_{2}}{\left(\frac{2r_{e}}{b}\right)}^{n_{3}}{\left(\frac{f_{30}}{f_{co}}\right)}^{n_{4}}{\left(\frac{h}{b}\right)}^{n_{5}}{\left(\frac{\varepsilon_{f}}{\varepsilon_{co}}\right)}^{n_{6}}$$

(14) $$\frac{\varepsilon_{cu}}{\varepsilon_{co}}=m_{1}+m_{2}{\left(\frac{E_{l}}{E_{c}}\right)}^{m_{3}}{\left(\frac{f_{30}}{f_{co}}\right)}^{m_{4}}\left(m_{5}\frac{2r_{e}}{b}+m_{6}\right){\left(\frac{h}{b}\right)}^{m_{7}}{\left(\frac{\varepsilon_{f}}{\varepsilon_{co}}\right)}^{m_{8}}$$

In Equations (13) and (14), *r_e_* is introduced as the equivalent corner radius. As discussed above, the elliptical cross-section can be considered a rectangular column with a special corner radius for calculation. When the cross-section is an ellipse, *r_e_* can be taken as the special equivalent value. Otherwise, *r_e_* is equal to the real corner ratio. Unknown coefficients $n_{1}-n_{6}$, $m_{1}-m_{8}$ and α and β in Equation (8) can be determined by the nonlinear numerical regression methodology using all of the data from the database in Table 2. For elliptical cross-sections, Equation (8) is substituted into Equations (13) and (14). Eventually, Equations (13) and (14) can be written as:

(15) $$\frac{f_{cc}}{f_{co}}=1+8.34{\left(\frac{E_{l}}{E_{c}}\right)}^{1.03}{\left(\frac{2r_{e}}{b}\right)}^{0.81}{\left(\frac{f_{30}}{f_{co}}\right)}^{0.54}{\left(\frac{h}{b}\right)}^{-1.9}{\left(\frac{\varepsilon_{f}}{\varepsilon_{co}}\right)}^{0.82}$$

(16) $$\frac{\varepsilon_{cu}}{\varepsilon_{co}}=1.75+9.45{\left(\frac{E_{l}}{E_{c}}\right)}^{0.68}\left(0.54\frac{2r_{e}}{b}+0.46\right){\left(\frac{f_{30}}{f_{co}}\right)}^{0.79}{\left(\frac{h}{b}\right)}^{-0.64}{\left(\frac{\varepsilon_{f}}{\varepsilon_{co}}\right)}^{1.14}$$

When the confinement stiffness ratio $\frac{E_{l}}{E_{c}}$ is equal to zero, the column is unconfined, and the values of Equations (15) and (16) are equal to the ultimate stress and strain of unconfined concrete. If $\frac{2r_{e}}{b}$ = 1 and $\frac{h}{b}$ = 1, the column has a circular cross-section; if $\frac{2r_{e}}{b}$ ≠ 1 and $\frac{h}{b}$ = 1, the cross-sectional shape is square; if $\frac{2r_{e}}{b}$ ≠ 1 and $\frac{h}{b}$ ≠ 1, the column has a rectangular cross-section. When Equations (15) and (16) are applied to circular, square and rectangular cross-sections, the equivalent corner radius *r_e_* is the real corner radius *r*.

For the elliptical cross-section, after the regression analysis above, α and β in Equation (8) can be easily determined to be 0.61 and 1.04, respectively. Moreover, the β value is quite close to one, so Equation (8) can be rewritten linearly as:

(17) $$\frac{2r_{e}}{b}=1-0.61(\frac{h}{b}-1)\;1\leq \frac{h}{b}\leq 2.64$$

**Figure 7:**
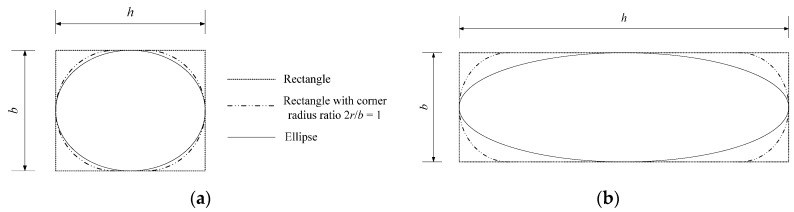
Elliptical cases. (**a**) *h ≈ b*; (**b**) *h >>* *b*.

**Figure 8:**
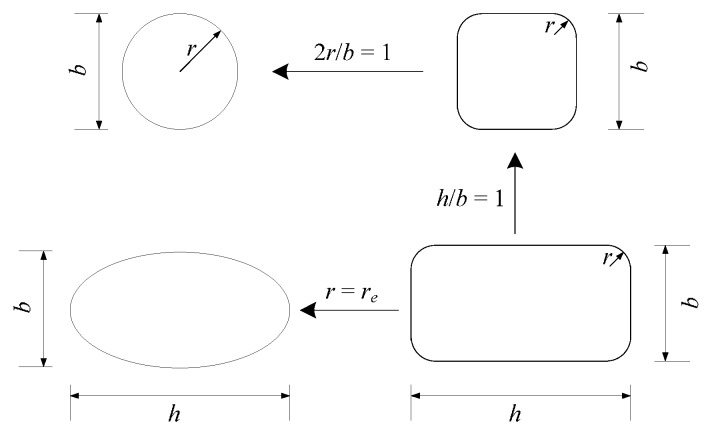
Cross-sectional unification.

When *h ≈ b* or *h*/*b* ≈ 1, the ellipse is close to a circle (Figure 7a), as is the ground rectangular section with 2*r*/*b* = 1. In this case, the confinement efficiencies should be very similar between two such cross-sections, which means that 2*r_e_*/*b* is close to one, as well. However, if *h >>* *b*, the corner at the major axis end is quite sharp, as shown in Figure 7b, which can cut the FRP much more easily. In this case, the confinement effect by FRP can be ignored. From Equation (17), 2*r_e_*/*b* = 0 when *h*/*b* = 2.64; that is, the FRP confinement can be ignored if *h*/*b* ≥ 2.64. This conclusion agrees with that of Campione and Fossetti [61]. Campione and Fossetti [61] noted that confinement efficiency could be ignored when the aspect ratio of the elliptical cross-section is greater than 2.6. Consequently, the unification of cross-sections is illustrated in Figure 8.

In Equation (7) and Figure 3, *E*_2_ is the slope of the asymptotic line of the hardening curve in the stress-strain relationship of concrete highly confined by FRP. According to its definition and conclusions in the existing investigations [14,19,28], the function of *E*_2_ uses the following equation.

(18) $$E_{2}=\frac{f_{cc}-f_{o}}{\varepsilon_{cu}}=\frac{f_{cc}-f_{co}}{\varepsilon_{cu}}$$

**Figure 9:**
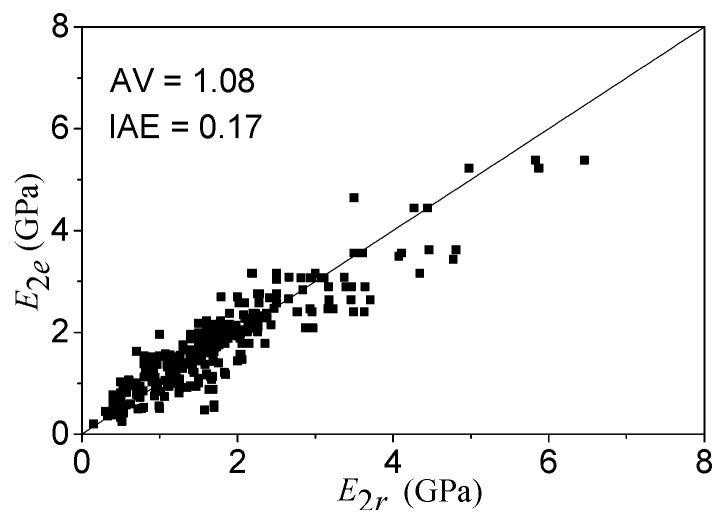
The performance of the *E*_2_ model.

In Equation (18), the parameters of *f_cc_* and ε*_cu_* use the functions of Equations (15) and (16), respectively. After regression, the *E*_2_ values of the theoretical (*E*_2*r*_) and experimental (*E*_2*e*_) results are compared, as shown in Figure 9. The evaluation index values of *AV* and *IAE* are 1.08 and 0.17, respectively.

## 5. Model Performance

### 5.1. Performance of the Ultimate Strain and Stress Model

**Figure 10:**
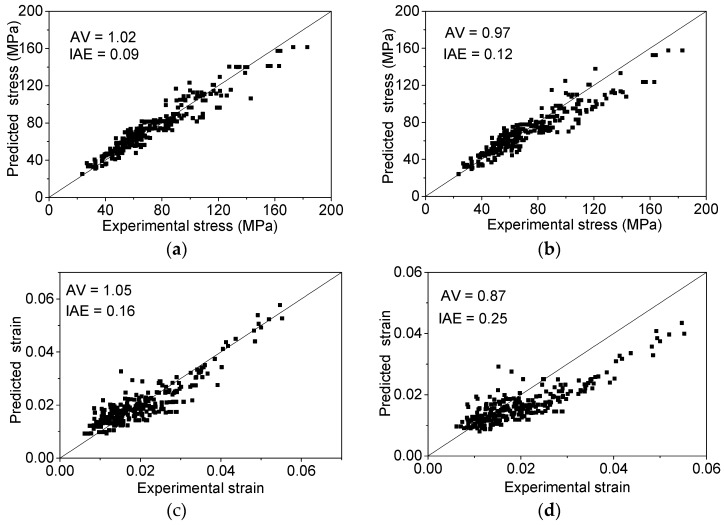
The performance of the ultimate stress and strain model for circular, square and rectangular columns. (**a**) Proposed ultimate stress model; (**b**) Wei and Wu’s ultimate stress model [19]; (**c**) proposed ultimate strain model; (**d**) Wei and Wu’s ultimate strain model [19].

After the evaluation and comparison of circular, square and rectangular columns, it is reported that Wei and Wu’s ultimate stress and strain model has better performance than other existing models [60]. Therefore, the models by Wei and Wu [19] are selected for comparison with the proposed models. The performances, with evaluation indexes values (*AV* and *IAE*), of the proposed ultimate models (Equations (15) and (16)) and Wei and Wu’s models for FRP-confined circular, square and rectangular columns are shown in Figure 10. The theoretical calculated results are compared to the experimental data in Figure 10. The *AV* and *IAE* index values show that the proposed models have better performance.

**Figure 11:**
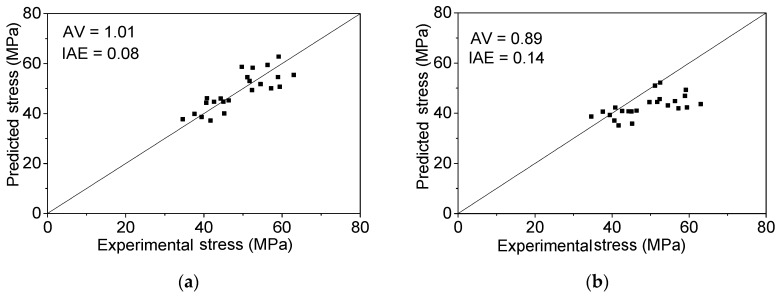

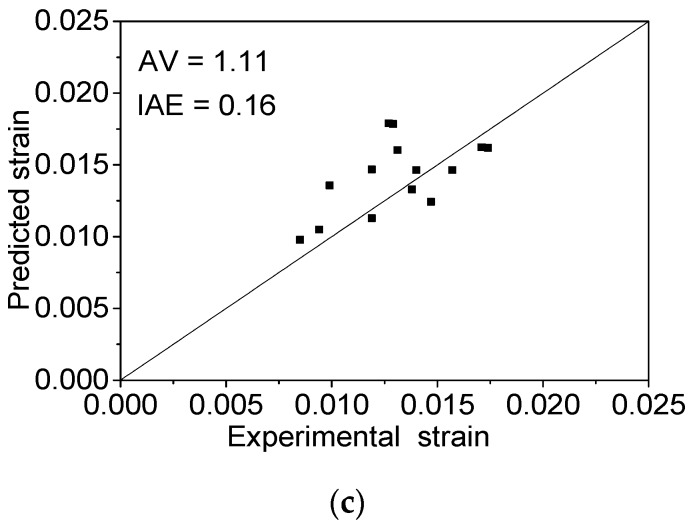
The performance of the ultimate stress and strain model for FRP confined elliptical columns. (**a**) Proposed ultimate stress model; (**b**) Teng and Lam’s ultimate stress model [32]; (**c**) proposed ultimate strain model.

The ultimate stress and strain for the FRP-confined elliptical column can be calculated by substituting Equation (17) into Equations (15) and (16). The performances, as well as the evaluation indexes values of the proposed ultimate stress model and Teng and Lam’s model [32] are illustrated in Figure 11a,b. The proposed ultimate stress model is more accurate according to the two index values. Because there are no existing ultimate strain models for the FRP-confined elliptical concrete column, only the proposed ultimate strain model is evaluated in Figure 11c with 1.11 and 0.16 for the *AV* and *IAE* values, respectively.

### 5.2. Performance of the Stress-Strain Relationship Model

**Figure 12:**
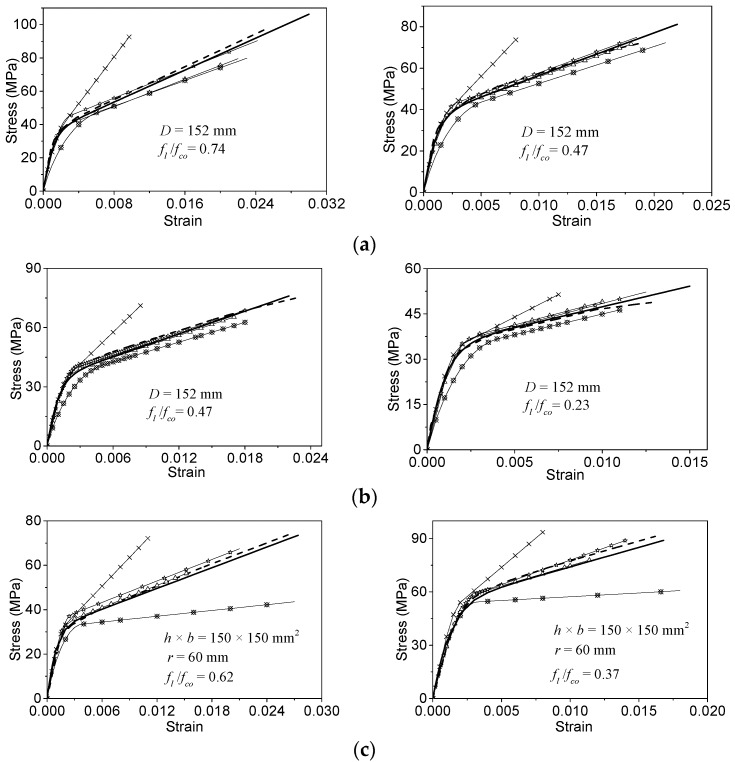

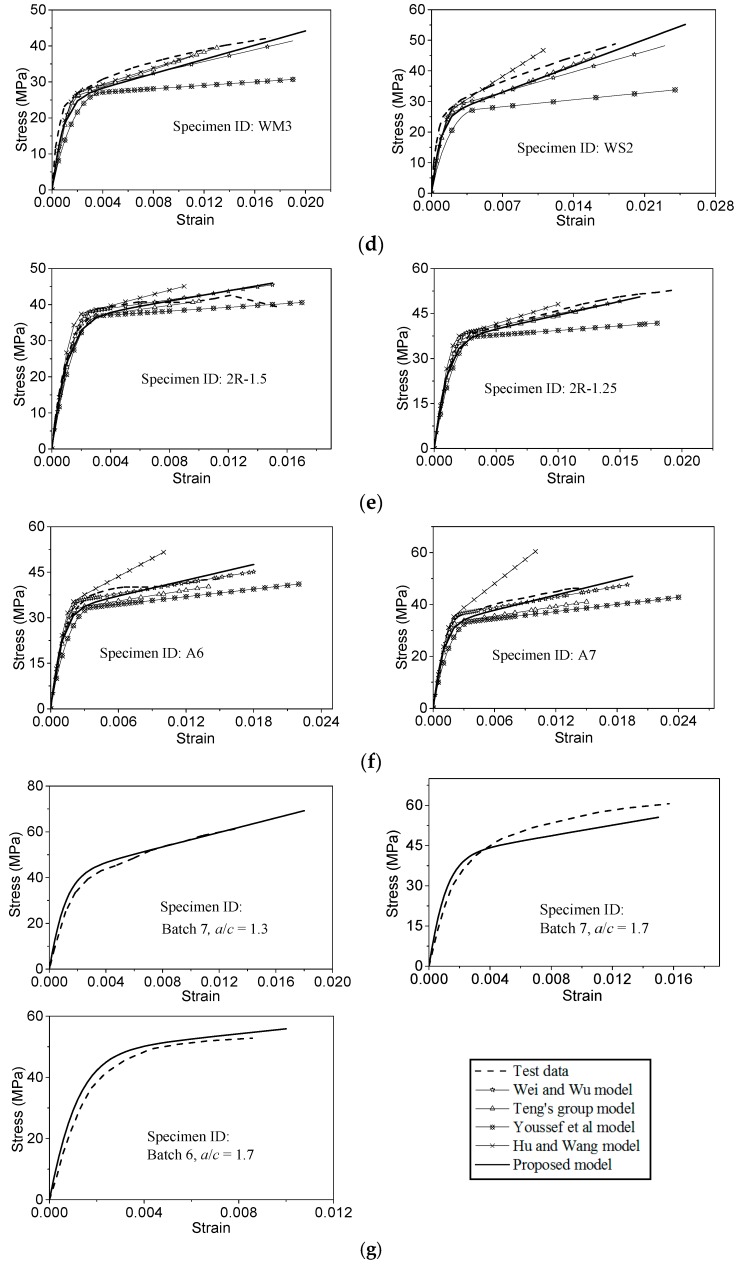
The performance of the proposed model for different cross-sectional columns. (**a**) Test results from Lam and Teng for circular columns [37]; (**b**) test results from Xiao and Wu for circular columns [35]; (**c**) test results from Wang and Wu for square columns [62]; (**d**) test results from Masia *et al.* for square columns [46]; (**e**) test results from Wu and Wei for rectangular columns [63]; (**f**) test results from Abbasnia and Ziaadiny for rectangular columns [50]; (**g**) test results from Stefano for elliptical columns [39].

Some selected experimental stress-strain curves collected from the literature [35,37,39,46,50,62,63] are used to evaluate the performances of the different stress-strain models. The experimental curves cover four cross-sections: circular (Figure 12a,b); square (Figure 12c,d); rectangular (Figure 12e,f); and elliptical (Figure 12g). The performances of both the proposed and other stress-strain models are shown in Figure 12. The original specimen IDs (if available) have been marked in the corresponding figures.

In terms of FRP-confined circular, square and rectangular columns, the predicted stress-strain curves from Hu and Wang’s model are obviously higher than the experimental results. The stress values calculated from Youssef *et al.*’s model are lower than the test data. The model by Teng’s group has better performance for the stress-strain curves than Hu and Wang’s model and Youssef *et al.*’s model. Moreover, Wei and Wu’s model has similar performance to the proposed model, but underestimates the ultimate stress and strain values. The proposed model shows more agreement with the experimental curves.

For the FRP-confined elliptical concrete specimens, only the proposed models can be evaluated (see Figure 12g). The proposed stress-strain model can predict the experimental cases well.

## 6. Conclusions

This work studied the cross-sectional unification of the stress-strain relationship for concrete under high FRP confinement. This work solved the problem that engineers face in having to choose different theoretical models to calculate FRP confinement for different cross-sectional concrete columns. The following conclusions can be drawn:

(1) The equivalent corner radius ratio (2*r_e_*/*b*) was introduced to transform the elliptical cross-section into a rectangular cross-section. The elliptical cross-section can be considered as a rectangular cross-section with a special corner radius ratio.
(2) Based on the equivalent confinement efficiency, the relationship between the ellipse and rectangle is obtained. A simple model of the equivalent corner radius ratio for the ellipse is proposed.
(3) Compared to other models and test data, the proposed model has better performance of ultimate stress and strain.
(4) According to the database, a unified stress-strain model is proposed for the FRP-confined different cross-sectional concrete columns. The advantage of this model is that it can predict the stress-strain relationship for FRP-confined circular, square, rectangular and elliptical columns. Compared to other models and experimental data, the proposed models in this paper show better agreement with the experimental data.

## Figures and Tables

**Table 1:** The details of the existing models.

Ref.	Model	Cross-section ^#^	Supplementary notation
Teng’s group [14,15,32,33]	$f_{cc}/f_{co}=1+k\left(f_{l}/f_{co}\right)$; $\varepsilon_{cc}/\varepsilon_{co}=1.75+5.53\left(f_{l}/f_{co}\right){\left(\varepsilon_{f}/\varepsilon_{co}\right)}^{0.45}$	C	*k* = 2 in [33] and 3.3 in [14]; ε*_f_* is the ultimate strain of FRP; ε*_co_* is the peak strain of plain concrete; *f_l_* is the confinement pressure; *r* is the corner radius; *h* is the length of the longer side of the rectangle; *b* is the length of the shorter side of the rectangle or the side of the square; *a* and *c* are the lengths of major and minor axis, respectively.
	$f_{cc}/f_{co}=1+3.3k_{s1}f_{l}/f_{co}$; $\varepsilon_{cc}/\varepsilon_{co}=1.75+12k_{s2}\left(f_{l}/f_{co}\right){\left(\varepsilon_{f}/\varepsilon_{co}\right)}^{0.45}$; $k_{s1}={\left(\frac{b}{h}\right)}^{2}\left(1-\frac{\left(b/h\right){\left(h-2r\right)}^{2}+\left(h/b\right){\left(h-2r\right)}^{2}}{3\left(bh-\left(4-\pi \right)r^{2}\right)}\right)$; $k_{s2}={\left(\frac{h}{b}\right)}^{0.5}\left(1-\frac{\left(b/h\right){\left(h-2r\right)}^{2}+\left(h/b\right){\left(h-2r\right)}^{2}}{3\left(bh-\left(4-\pi \right)r^{2}\right)}\right)$	R	
	$f_{cc}/f_{co}=1+2{\left(c/a\right)}^{2}\left(f_{l}/f_{co}\right)$	E	
Youssef *et al.* [28]	$f_{o}/f_{co}=1+3{\left(\rho_{f}E_{f}\varepsilon_{jt}/f_{co}\right)}^{1.25}$; $f_{cc}/f_{co}=1+2.25{\left(f_{l}/f_{\text{co}}\right)}^{1.25}$; $\varepsilon_{o}=0.002748+0.1169{\left(\rho_{f}E_{f}\varepsilon_{jt}/f_{co}\right)}^{6/7}{\left(f_{frp}/E_{f}\right)}^{1/2}$; $\varepsilon_{cu}=0.003368+0.2590\left(f_{l}/f_{co}\right){\left(f_{frp}/E_{frp}\right)}^{0.5}$	C	ρ*_f_* is the volume ratio of the FRP; *E_f_* is the elastic modulus of the FRP; ε*_jt_* is the FRP strain at the transition from the first to the second region, which is equal to 0.002; and *f_frp_* is the tensile strength of FRP.
	$f_{o}/f_{co}=1+1.135{\left(\rho_{f}E_{f}\varepsilon_{jt}/f_{co}\right)}^{1.25}$; $f_{cc}/f_{co}=0.5+1.225{\left(f_{l}/f_{\text{co}}\right)}^{0.6}$; $\varepsilon_{o}=0.002+0.0775{\left(\rho_{f}E_{f}\varepsilon_{jt}/f_{co}\right)}^{6/7}{\left(f_{frp}/E_{f}\right)}^{1/2}$; $\varepsilon_{cu}=0.004325+0.2625\left(f_{l}/f_{co}\right){\left(f_{frp}/E_{frp}\right)}^{0.5}$	R	
Hu and Wang [13]	$f_{ct}=\frac{f_{co}}{1-\frac{4\nu_{ct}k_{s}tE_{f}\varepsilon_{co}\left(\nu_{ct}-v_{f}\right)}{2tE_{f}\varepsilon_{co}\left(1+\nu_{ct}\right)\left(1-2\nu_{ct}\right)+bf_{co}\left(1-\nu_{f}^{2}\right)}}$; $\varepsilon_{ct}=\frac{f_{ct}\varepsilon_{co}}{f_{co}}$; $f_{cc}/f_{co}=0.5+2.7k_{s}^{2.24}{\left(f_{l}/f_{co}\right)}^{0.68}$; $\nu_{ct}=1-0.0025\left(f_{co}-20\right)$; $\varepsilon_{cu}=\left[A_{f}k_{s}f_{frp}^{2}+E_{f}A_{c}\left(\varepsilon_{sp}f_{co}+\varepsilon_{co}f_{cc}\right)\right]/\left[E_{f}A_{c}\left(f_{co}+f_{cc}\right)\right]$; $k_{s}=1-\frac{\left(b/h\right){\left(h-2r\right)}^{2}+\left(h/b\right){\left(h-2r\right)}^{2}}{3\left(bh-\left(4-\pi \right)r^{2}\right)}$	C&R	ν*_ct_* is Poisson’s ratio for the turning point; *t* is the thickness of FRP; *E_f_* is the tensile elastic modulus of FRP; ν*_f_* is Poisson’s ratio for FRP; *b* is the diameter of the circular cross-section or the width of the rectangular and or square cross-section; *A_f_* and *A_c_* are the areas of the FRP and concrete, respectively.
Wei and Wu [19].	$f_{o}=f_{co}+0.43{\left(\frac{2r}{b}\right)}^{0.68}{\left(\frac{h}{b}\right)}^{-1}f_{l}$; $\frac{f_{cc}}{f_{co}}=1+2.2{\left(\frac{2r}{b}\right)}^{0.72}{\left(\frac{f_{l}}{f_{co}}\right)}^{0.94}{\left(\frac{h}{b}\right)}^{-1.9}$; $E_{2}=\frac{f_{cc}-f_{o}}{\varepsilon_{cu}-\varepsilon_{o}}$; $\varepsilon_{o}=\left[\left(f_{o}+f_{cu}+E_{c}\varepsilon_{cu}\right)-\sqrt{{\left(f_{o}+f_{cc}+E_{c}\varepsilon_{cu}\right)}^{2}-8f_{o}E_{c}\varepsilon_{cu}}\right]/2E_{c}$; $\frac{\varepsilon_{cu}}{\varepsilon_{co}}=1.75+12{\left(\frac{f_{l}}{f_{co}}\right)}^{0.75}{\left(\frac{f_{30}}{f_{co}}\right)}^{0.62}\left(0.36\frac{2r}{b}+0.64\right){\left(\frac{h}{b}\right)}^{-0.3}$	C&R	*f*_o_ and ε*_o_* are the transitional stress and strain of confined concrete, respectively; *f_cc_* and ε*_cu_* are the ultimate stress and ultimate strain of confined concrete, respectively; and *f*_30_ is the concrete strength of unconfined grade C30 concrete, which is equal to 30 MPa.

^#^ C denotes circular; E denotes elliptical; R denotes rectangular.

**Table 2:** The summary of the details of the stress-strain curves.

Reference	Specimen No.	Section	Specimen size	*f_co_* (MPa)	FRP type	*f_frp_* (MPa)	*t_frp_* (mm)
		Type ^#^	(mm) ^				
Xiao and Wu [35]	17	C	152 × 305	33.7–55.2	CFRP	1,577	0.38–1.14
Karabinis and Rousakis [36]	7	C	200 × 320	35.7–38.5	CFRP	3,720	0.117–0.351
Lam and Teng [37]	13	C	152 × 305	34.3–38.5	CFRP,	4,203	0.165–0.495
					GFRP	490	1.27–2.54
Almusallam [38]	5	C	150 × 300	48–60	GFRP	540	1.3–1.9
Lam *et al.* [44]	6	C	152 × 305	39, 41	CFRP	3,754; 3,800	0.165, 0.33
Stefano Casalboni [39]	5	C	200 × 400	32.6–47.8	CFRP	3800	0.171–0.342
Wang [40]	12	C	150 × 300	30.9, 52.1	CFRP	3,788; 4,364	0.165–0.33
Cui and Sheikh [41]	58	C	150 × 300	45.6–85.6	CFRP, GFRP	849–3,648	0.111–3
Akogbe *et al.* [42]	2	C	100 × 200; 200 × 400	33.8	CFRP	3,248	0.167–0.334
Cao *et al.* [25]	11	C	150 × 300	25–60	CFRP	4,192	0.0495–0.33
Wu and Jiang [45]	33	C	150 × 300	20.6–36.7	CFRP	4,441	0.167–0.835
Wu *et al.* [43]	12	C	150 × 300	32–53	CFRP	4,192	0.167–0.334
Teng and Lam [32]	9	E	168 × 132 × 600, 195 × 115 × 600, 238 × 95 × 600	36.6–39.0	CFRP	3,983; 3,824	0.165, 0.22
Stefano Casalboni [39]	14	E	200 × 100 × 400, 200 × 120 × 400, 200 × 155 × 400.	32.6–47.8	CFRP	3,800	0.171–0.342
Rochette and Labossiere [51]	2	S	152 × 152 × 500, *r*: 25,38	35.8, 42	CFRP	1,265	0.9, 1.2
Lam and Teng [15]	6	S	150 × 150 × 600, *r*: 15, 25	24, 33.7	CFRP	4,519	0.165–0.495
Masia *et al.* [46]	6	S	(100–1500) × (100–150) × (300–450) *r*: 25	23.8–24	CFRP	3,500	0.26
Wang [40]	27	S	150 × 150 × 300, *r*: 30–60	30.9, 52.1	CFRP	3,788; 4,364	0.165–0.33
Tao *et al.* [47]	4	S	150 × 150 × 450, *r*: 20, 35	22, 49.5	CFRP	4,200; 4,470	0.17, 0.34
Abbasnia *et al.* [6]	1	S	150 × 150 × 300, *r*: 42	30	CFRP	3,943.5	0.489
Wang *et al.* [48]	10	S	(100–400) × (100–400) × (300–1200) *r*: 10–45	24.4	CFRP	4,340	0.167–0.668
Wei [49]	5	S	150 × 300 × 300, *r*: 30	35.3	CFRP	4,192	0.167–0.334
Abbasnia and Ziaadiny [50]	7	S	150 × 150 × 300, *r*: 13.6–42	32–51.5	CFRP	3,943.5	0.352
Lam and Teng [15]	1	R	150 × 225 × 600, *r*: 25	41.5	CFRP	4,519	0.66
Chaallal *et al.* [52]	4	R	108 × 165, *r*: 25.4	25.1	CFRP	3,650	0.17
Tao *et al.* [47]	4	R	150 × 230 × 450,	19.5, 22	CFRP	4,470	0.34
			150 × 300 × 450, *r*: 20–50				
Abbasnia *et al.* [6]	2	R	(90, 120) × 180 × 300, *r*: 25.2–33.6	30	CFRP	3,943.5	0.489
Wei [49]	6	R	150 × 188 × 300,	35.3	CFRP	4,192	0.167–0.335
			150 × 225 × 300, *r*: 30				
Abbasnia and Ziaadiny [50]	7	R	90 × 180 × 300, 120 × 180 × 300, *r*: 18.1–34.5	32–51.6	CFRP	3,943.5	0.352

^#^ C denotes circular; E denotes elliptical; S denotes square; R denotes rectangular. ^^^ *b* × *h* × *L*, *r* (breadth × depth × length, corner radius) for rectangular and square columns; *d* × *L* (diameter × length for circular columns); *a* × *b* × *L* (major axis × minor axis × length) for elliptical columns.
